# Is there a reason to challenge our current practice in children’s forearm fractures?

**DOI:** 10.1080/17453674.2020.1854505

**Published:** 2020-12-10

**Authors:** L Stefan Lohmander, Ian A Harris

**Affiliations:** a Department of Clinical Sciences Lund, Orthopaedics, Lund University, Sweden.;; b South Western Sydney Clinical School, University of New South Wales, and Department of Orthopaedic Surgery, Liverpool Hospital, Liverpool, NSW, Australia.

*Sir,—*The paper by Laaksonen et al. (2020) in this issue of *Acta Orthopaedica* shows practice variation and possible overtreatment for a common orthopedic presentation: completely displaced (including overriding) distal radius fractures in children. While there is a considerable “grey zone” in treating these fractures, based on factors such as the age and sex of the child, and the proximity of the fracture to the wrist, there should be better agreement than has been demonstrated in this paper, and what many of us see in clinical practice.

We believe that the results presented by Laaksonen and colleagues raise several important questions about orthopedic practice, variation therein, and the management of these fractures in particular. Questions such as: “What is the cause of the variation?,” “Is better quality evidence for benefit and harms needed for the different treatments?,” and “How can the practice be brought in line with what is best for the patient?”

Practice variation occurs where clear guidance is not provided by high-quality evidence. Sometimes practice variation occurs even when high-quality evidence exists (Grove et al. 2016, Lohmander et al. 2016). For the fractures discussed here, there are no published low-risk-of-bias comparative trials that have compared cast-only treatment with closed reduction alone, or closed reduction and percutaneous fixation (Handoll et al. 2018, Zeng et al. 2018). Evidence from such trials would be helpful, and would need to cover the “grey zone” where most practice variation is likely to occur. For example, there may be little practice variation for patients aged less than 3 years, or those close to skeletal maturity, so inclusion of the ages in between and an analysis based on sex and fracture anatomy would be needed. Such a trial would ideally be randomized at an individual level but could also be done using cluster randomization of institutions with crossover, where all eligible patients at each participating institution would be included as part of routine care, first with one treatment, then with the comparator, over two time periods. This would allow fairer comparisons and better generalizability, and would aid with recruitment.

If the randomized trial design is deemed not feasible or acceptable, the next best alternative could be a prospective observational study, comparing 2 or more treatment alternatives. This would need to be as carefully designed and reported as a randomized trial, to provide a fair comparison between study groups, but without the randomization. With this design, however, risk of bias due to insufficient matching of study groups and to unknown factors remains high.

Related to the question of “what is best for the patient” is what the primary outcome of such a trial should ideally be. Active range of motion? Patient-reported pain and function? Patient-reported overall satisfaction or quality of life? Imaging-based outcome? For interventions regulated by the FDA and EMA, primary outcomes are to be clinically relevant, i.e., how a patient feels, functions, or survives. Blinding is a challenge in surgical trials, but many, perhaps most, endpoint assessments can be observer blinded.

We might argue that given that we do not have strong evidence in favor of more invasive management, the current default treatment should be cast immobilization alone, the assumption being similar benefit, less harm, and lower cost. Observational evidence to support treating these fractures without reduction and with excellent outcome was published several years ago (Do et al. 2003, Crawford et al. 2012). However, as the Laaksonen survey shows, surgeons are currently not inclined to treat these fractures without reduction.

Several reasons may contribute to this attitude. The bias towards more invasive management may be due to “defensive medicine” where doctors, because of unmotivated fear of poor outcome with nonoperative treatment, try to avoid possible future complaints. Another reason could be bias introduced due to the mentor–trainee guidance in surgical training. The silo environment of a highly specialized community that has not fully adopted the principles of evidence-based surgery may also contribute: “If your only tool is a hammer, you tend to treat everything as a nail.” Surgeons often tend to intervene when evidence is lacking—of operating until the evidence says otherwise—while the opposite should be the rule. A contributing factor that biases treatment towards more invasive options is that doctors, and patients, are susceptible to overestimating the benefits and underestimating the harms of their interventions (Hoffmann and Del Mar 2017). The particular case discussed here is likely no exception. Until further treatment studies have been published it seems prudent to recommend nonoperative treatment of these fractures, the burden of proof belonging to those who recommend operative treatment.

The practice of evidence-based surgery in orthopedics faces numerous obstacles (Grove et al. 2016, Robinson et al. 2019, Emara et al. 2020). The randomized controlled trial comparing treatment with a surgical procedure to a treatment without surgery, or even with placebo surgery, while increasing, remains a distinct minority in our field (Lim et al. 2014, Wartolowska et al. 2014, Beard et al. 2020, Harris et al. 2020, Skou et al. 2020).

We know from recent examples of orthopedic practice, such as arthroscopic surgery of the knee and shoulder for pain, and vertebroplasty for osteoporotic vertebral fractures, that even if enough scientific evidence were generated to guide practice in this area, the eventual practice change would be a gradual, behavioral one. There is often a lag of many years between evidence being published and a resulting change in practice. Surgeons, like most professions, are insular and look to each other for clues regarding acceptable behavior. While influential surgeons can change practice amongst peers, change still needs to come down to individual surgeons questioning their current beliefs and looking objectively at their current practices within the context of the practice of those around them.

The findings of Laaksonen and colleagues should provide a stimulus for low-risk-of-bias comparative studies in this area so that practice can be narrowed to a range that targets the best patient outcomes with the least harm and cost. One such trial was planned and a protocol published (Adrian et al. 2015), but no results appear to have been published. We are encouraged to note that the authors of the survey discussed here have initiated a randomized trial of “Casting Versus Percutaneous Pinning Treatment of Pediatric Overriding Distal Forearm Fractures” (https://clinicaltrials.gov/ct2/show/NCT04323410).

We call on surgeons to question their current practice, to participate in planned trials, and to be open to new evidence as it is presented. Evidence-based surgery differs from non-evidence-based surgery in that the former necessitates that judgments are consistent with underlying evidence, while the latter does not.

L Stefan LOHMANDER ^1^ and Ian A HARRIS ^2^ *^1^ Department of Clinical Sciences Lund, Orthopaedics, Lund University, Sweden.* *^2^ South Western Sydney Clinical School, University of New South Wales, and Department of Orthopaedic Surgery, Liverpool Hospital, Liverpool, NSW, Australia.* *Email:*stefan.lohmander@med.lu.se *Email:*iaharris1@gmail.com
